# When Nudges Don’t Budge: A Mixed Methods Study of Why EHR-Based Deprescribing Nudges Failed to Change Provider Behavior

**DOI:** 10.21203/rs.3.rs-7466262/v1

**Published:** 2025-09-23

**Authors:** Ratnalekha V. N. Viswanadham, Hayley M Belli, Tiffany Rose Martinez, Christina Wong, Saul Blecker, Andrea B Troxel, Devin M Mann

**Affiliations:** NYU Grossman School of Medicine: New York University School of Medicine; NYU Grossman School of Medicine: New York University School of Medicine; NYU Grossman School of Medicine: New York University School of Medicine; NYU Langone Medical Center: NYU Langone Health; NYU Grossman School of Medicine: New York University School of Medicine; NYU Grossman School of Medicine: New York University School of Medicine; NYU Grossman School of Medicine: New York University School of Medicine

**Keywords:** electronic health records, behavioral economics, implementation science, diabetes, nudge, choosing wisely, clinical decision support, mixed methods research

## Abstract

**Background:**

De-implementation—reducing low-value or harmful care—is critical but often difficult in practice. Nudges via clinical decision support (CDS) tools in electronic health records aim to promote guideline-concordant care, but their effectiveness is mixed. In a randomized trial, we tested CDS nudges to support deprescribing glycemic medications in older adults, aligned with Choosing Wisely guidelines. Despite prior success elsewhere, the intervention had limited impact. The current study evaluated potential reasons why the EHR-based nudges to encourage guideline-based, relaxed glycemic control for older adults with Type 2 Diabetes were not effective in influencing clinician behavior.

**Methods:**

We conducted a retrospective cohort analysis of EHR data from 67,412 alerts issued to clinicians, promoting different types of glycemic control, including reducing metformin, switching from non-metformin medications to metformin, and discontinuing medication. Comments left by providers on 779 of those firings were coded and thematically analyzed by two authors. Logistic and multinomial logistic regressions were performed to understand the contexts behind the lack of nudge effectiveness at the alert, encounter, patient, and physician levels.

**Results:**

Out of 67,412 alerts, providers commented in only 1.15% of cases. When they did, they were about 10.7% more likely to act on the alert, but comments were mostly negative (3.28 times more likely). Feedback highlighted three themes: disagreement with guidelines (most common), poor alert fit in workflow, and patient reluctance to change medications. Logistic regressions showed providers were less likely to act on alerts with multiple triggers and more likely to leave negative comments. Multinomial models linked rejection themes to patient and medication traits, noting less rejection related to workflow in patients with limited life expectancy. Disparities in engagement were found, with female providers, patients, and socially vulnerable individuals less likely to comment.

**Conclusion:**

These findings highlight barriers to de-implementation via CDS. Provider disagreement, misaligned alerts, and patient resistance hinder effectiveness. Low engagement and negative feedback suggest nudges alone may not change behavior without integration into routines. Engagement variation stresses the need for tailored strategies. Future work should refine nudge design to address complexity, align with provider roles, and include patient-centered approaches.

**Trial registration:**

The NYU School of Medicine Institutional Review Board (i17-01308) approved the trial, which has the clinicaltrials.gov ID NCT04181307 (https://clinicaltrials.gov/study/NCT04181307), with date of first record on November 26, 2019.

## INTRODUCTION

The de-implementation of healthcare practices—intentionally discontinuing ineffective or potentially harmful interventions—is essential to health services research, aiming to improve patient outcomes and optimize resource allocation. (1) Yet, despite its significance, de-implementation remains underutilized and undertheorized in clinical practice. (2) One main reason is the difficulty. De-implementation behavior is challenging because, in a larger context, convincing people to stop habitual behaviors is challenging. Unlike implementation, which often involves building motivation and creating systems for adopting new practices, de-implementation must overcome deeply embedded habits, institutional norms, and fears of unintended consequences. Getting individuals, especially providers with competing demands and limited time, to stop doing something is often more difficult than encouraging them to start a new behavior.

For example, in deprescribing, providers face multiple challenges that lead them to stick with usual care, potentially avoiding deprescribing decisions or failing to deprescribe medications. (3, 4) Significant time pressures may limit their ability to thoroughly evaluate the necessity of each medication and engage in detailed discussions about deprescribing with patients. Providers might worry about damaging rapport with patients, especially if the patient perceives deprescribing as a withdrawal of care. Providers may lack confidence in deprescribing guidelines due to concerns about the risks and liabilities associated with de-implementation for themselves and their patients, particularly regarding potential adverse outcomes. (5)

To mitigate these, clinical practice guidelines and educational campaigns have been developed to assuage anxiety and challenges of de-implementation. For example, the American Board of Internal Medicine launched the Choosing Wisely (CW) campaign, which focuses on reducing redundant health tests and treatments to improve healthcare quality. (5–7) The campaign has helped bring national attention to the overuse of certain services and promoted more thoughtful conversations between patients and providers. However, despite widespread dissemination, CW guidelines have not always led to measurable reductions in low-value care. This gap between awareness and behavioral change highlights the limitations of passive information dissemination and underscores the need for more active implementation strategies. (8)

However, because disseminating clinical guidelines through informational channels rarely changes practice behavior, implementation science research has sought more effective ways to raise awareness about de-implementation practices, such as deprescribing. (9) Nudges involve altering the choice architecture to guide individuals toward specific choices while maintaining their autonomy in decision-making.(10) When applied through clinical decision support (CDS) within electronic health records (EHRs), nudges have become increasingly popular for promoting clinical guidelines. CDS proves effective by offering timely and relevant guidance at the moment of decision-making. (11) For example, it can assist when a provider enters a medication order or composes a message, seamlessly integrating recommendations into their workflow. (12, 13) Implementation science research has therefore sought more effective mechanisms to bridge this gap, particularly in high-volume, fast-paced clinical settings. CDS tools, for example, can flag medication orders that may be inappropriate based on patient characteristics or prompt clinicians with tailored recommendations during chart review, order entry, or patient messaging workflows.

While some research has reported that nudging in healthcare can effectively change behavior, a growing realization is emerging that not all interventions achieve the desired impact, prompting a need to explore contexts where nudges fall short. (14, 15) Contextual factors such as the timing of the alert, competing clinical priorities, and trust in the CDS system all shape how nudges are received and acted upon. Research has shown that multicomponent interventions targeting clinicians to effectively implement care guidelines change clinical practices, thereby reducing the delivery of low-value patient care. (8) However, the specific mechanisms by which nudges succeed or fail in EHRs remain poorly understood.

A study from our group employed a randomized controlled trial across a large academic health system to assess the effectiveness of nudges via clinical decision support in implementing CW guidelines that promote less aggressive glycemic control in pharmacological therapies for geriatric populations with type 2 diabetes (DM2).(16) (17–19) These guidelines reflect a growing consensus that tight glycemic control in older adults may increase the risk of hypoglycemia and other adverse outcomes, particularly in those with comorbidities, cognitive impairment, or limited life expectancy. Results from the RCT showed that the nudges based on these guidelines did not significantly change provider behavior when deprescribing glycemic control medications for older patients. (16)

Given that nudges have shown mixed effects on changing behavior(20), it is crucial to understand why specific nudges within a study led to null results and what contextual factors contributed to these outcomes. Fortunately, one of the CDS tools integrated into the EHR included an option for providers to provide a reason for their acknowledgment, which offers valuable insights into why providers did not act on the CDS recommendations. Previous literature has shown that incorporating provider feedback in the EHR can significantly enhance CDS design.(21) Moreover, the ability to collect contextual insights at the point of care represents a novel and underused method for evaluating and improving CDS systems within a learning health system.

This research aims to understand the limited success of implementing nudges in practice within a large, embedded, pragmatic clinical trial. By systematically examining instances when anticipated behavioral changes failed to materialize during de-implementation, we add to the existing body of knowledge on nudges and healthcare. In doing so, we aim to refine strategies for discontinuing healthcare practices that may compromise patient well-being, enhance the design of CDS interventions, and strengthen the evidence base on when and how nudges can work effectively in real-world clinical settings.

## METHODS

### Study Aim, Design, and Setting

This study employed a mixed-methods approach to evaluate and interpret clinician comments retrospectively, identifying why clinicians agreed or disagreed with proposed care guidelines for de-prescribing DM2 medications in older adult patients. A mixed-methods approach was necessary to capture both the qualitative insights from provider comments regarding disagreements with the CDS nudge intervention and the quantitative insights into the associations between the CDS and sentiments. This approach enables us to understand not only the effectiveness of the alerts but also the contextual factors that influence provider decisions and barriers to implementation. This study adheres to the GRAMMS checklist for mixed methods research, ensuring rigorous reporting of both the thematic analysis of provider comments and the quantitative analysis of alert firings. (22, 23) The study was conducted within the New York University Langone Health (NYULH) EHR and Epic system.

**Figure 1:**
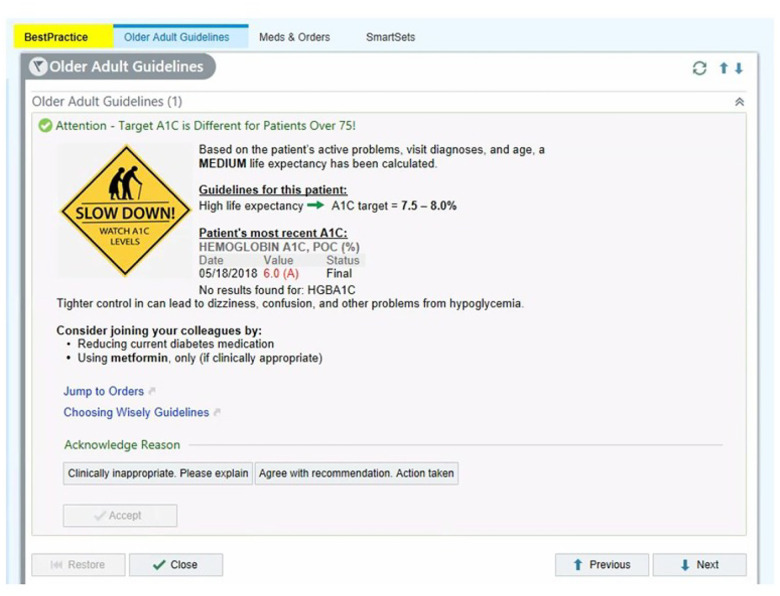
An example of an alert (formerly called a Best Practice Advisory and now Our Practice Advisory) to guide providers to prescribe medication appropriately.

Details about the randomized control trial have been published. (19) In summary, a set of six CDS tools, designed using principles of behavioral economics and developed through a user-centered design process, nudged providers to ease the administration of medications aimed at glycemic control for geriatric patients with DM2. These CDSs served as nudges because they guided provider decision-making without restricting providers’ autonomy regarding patient care (i.e., providers could override the suggestions made by the CDS). One type of clinical decision support implemented in the RCT was a built-in reminder in the Epic EHR system known as an “our practice alert” (OPA). The CW guidelines were promoted within the Epic EHR through nine unique OPAs that were designed based on a combination of recommended pharmacological adjustments to glycemic control (switching from a non-Metformin medication to Metformin, lowering the non-Metformin dose, or lowering the Metformin dose) and the patient’s life expectancy (high, medium, and low). The EHR data recorded whether the provider interacted with the OPA and, if so, the nature of their engagement (e.g., whether the OPA was ignored, if the provider acknowledged the OPA but took no action, or if the provider acknowledged the OPA and followed through with the suggestion). Additionally, a provider who engaged with the OPA could leave comments justifying their choice of action regarding the OPA. An example of such an OPA is shown in Figure 1.

### Study Population

The study population consisted of physicians and advanced practice providers at NYULH between December 2016 and July 2023 who could act upon OPAs in the EHR regarding medication titration for DM2 during patient encounters.

### Data and Coding

Data were pulled from the firing of the OPAs between December 22, 2016, and July 28, 2023, with an SQL query from the EHR’s data warehouse. Although the RCT testing the package of nudges was conducted between March 9, 2020, and September 8, 2021, data were collected from OPA firings from the pilot period before the RCT and after the RCT to account for the acute impact of the COVID-19 pandemic on healthcare delivery, which decreased healthcare utilization. (24) The data of interest from the OPAs were the acknowledgment comments left by providers through the OPAs.

Covariates collected include patient-level demographics (age, self-identified race and ethnicity, self-identified sex, and BMI), provider-level variables (self-identified gender, type of provider, and provider specialty), and encounter-level variables (the number of firings of other OPAs during the patient’s encounter with the provider as a proxy of provider busyness, the number of unique encounters, and the type of encounter). Race and ethnicity categorizations of patients were based on the U.S. Census Bureau’s categorizations of race and ethnicity. (25)

RVNV and HMB independently coded physician comments and then convened to compare their coding. This led to the collaborative determination of primary themes and associated sub-themes. Comments could have been categorized with more than one main theme. The coded themes were classified as either positive (when providers agreed and acted in accordance with the OPA) or negative (when providers did not agree with the OPA).

### Statistical Analyses

Descriptive statistics were collected for the study population to understand the distributions of patient, provider, OPA, and encounter-level covariates. They were also collected for the types of actions taken with the OPAs and the number of comments within the identified themes and sub-themes.

Logistic regression was conducted to evaluate whether there were associations between a provider not responding to an OPA’s characteristics (a representation of provider engagement with the OPA) and encounter characteristics (the context in which OPAs are not responded to). Next, a multinomial logistic test was conducted to determine whether an identified rejection theme (versus an acceptance of the OPA) was associated with the type of glycemic control rejected or a patient’s mortality, given that the glycemic controls can be associated with varying provider preferences for glycemic control and that a provider may feel differently. The null hypothesis for these regressions would be that no significant variation existed between why a provider acknowledged a comment and the medication recommendation. The alternate hypothesis implies that the identified themes for rejecting the nudges were significantly associated with different suggestions.

Representativeness checks were conducted to check whether commenting behavior was notable for a particular provider or patient population during the RCT period. First, we used logistic regression to determine whether, for OPAs acknowledged, comments on OPAs were left by a representative population of physicians and a representative population of patients in the study. Second, we used a multinomial test to compare whether the distribution of themes in comments left on OPAs during the RCT period was representative of the distribution of themes in comments left during the entire period of the OPAs’ firings, given that the RCT started during the acute COVID-19 pandemic. If the statistical test results fail to reject the null hypothesis (i.e., no statistically significant coefficients in the regressions), then the population of comments from the RCT period would be comparable to those left outside the RCT period.

All coefficients were converted into odds ratios for ease of interpretation. Analyses were conducted in RStudio version 4.3.2.

## RESULTS

### Descriptive Statistics

Table 1 provides descriptive statistics of the patient, provider, encounter, and OPA characteristics. Of the 67,412 times the OPAs were fired between December 22, 2016, and July 28, 2023, providers left 779 comments (1.15%). Of the 25,045 times the OPAs were fired between March 9, 2020, and September 8, 2021 (the RCT period), providers left 215 comments (0.85%).

### Emergent Themes from Comments

Of the comments provided, 82 providers in 763 encounters acted based on the OPA’s recommendation, while 364 providers in 39132 encounters did not, indicating that providers selectively commented on OPAs. This suggests that while most nudges did not lead to deprescribing behavior, a subset of providers was motivated to justify or explain their clinical reasoning, providing a unique window into real-world decision-making. Three major themes were identified among the reasons providers did not utilize the OPA, with some comments meeting multiple major themes. Table 2 provides counts of emergent themes, frequency statistics, and representative comments from those left in the OPAs during their activation.

The most frequent theme in the comments was that the providers disagreed with the CW guidelines (N = 308 comments). This disagreement often reflected clinical judgment that diverged from guideline recommendations, underscoring tensions between standardized guidelines and individualized care. In most cases, the provider would determine that the patient had no symptoms of hypoglycemia or issues associated with tight glycemic control and appeared well (N = 160 comments). These comments reflected a perception that the risks of overtreatment were low or not applicable to the patient in question. The second sub-theme that emerged was that the clinician disagreed with the nudge or guidelines (N = 78 comments). These comments more explicitly questioned the relevance, appropriateness, or utility of the CDS tool itself. The third sub-theme was that the provider had other clinical reasons not to act per the OPA’s suggestion, such as the patient having a comorbidity that would not work with the suggestion (e.g., CKD, anemia, or CAD) or a clinical history of not responding well to the OPA’s suggestion (N = 76 comments). In such cases, clinicians provided nuanced clinical reasoning that accounted for complexities not captured by the CDS logic. Fourth, controlling the patient’s A1C was a secondary objective to their current prescription (N = 9 comments). This indicates that glycemic control may have been deprioritized in favor of managing more immediate or pressing health concerns.

The second major theme was that nudges were placed in the wrong location within the clinical workflow, making it difficult for providers to take meaningful action (N = 203 comments). This highlights the importance of aligning CDS delivery with key decision-making moments in clinical care. Most commonly, the patient’s glycemic control was often not the responsibility of the provider who received the OPA (e.g., the patient’s primary care provider or endocrinologist), so the glycemic control was not the provider’s responsibility (N = 125 comments). This misalignment likely contributed to alert fatigue or inaction, as the recipient of the alert lacked the contextual authority or responsibility to act. The other subtheme of perceived incorrect placement of the comments was that a provider would want to await a new lab result to reevaluate a patient’s current prescription. The provider also noted that the discussion should be deferred until the next appointment with the patient, indicating that more time was needed to consider the suggestion (N = 89 comments). These comments reflect temporal misalignment—nudges were delivered before the provider felt they had sufficient information or opportunity to act, suggesting the need for more adaptive or anticipatory CDS timing.

The third major theme that emerged was that patients needed encouragement to change their medications (N = 69 comments). In these cases, providers recognized the appropriateness of the CDS recommendation but described barriers rooted in patient preferences or resistance to the recommendation. Representative comments on this theme included “patient refuses to reduce meds,” “Patient has been reluctant to stop his medication despite the guidelines which have been discussed, and he clearly understands; however, since he is tolerating his regimen well with no hypoglycemic episodes, he asks to continue treatment,” and “Patient wants to stay on medication optimize glycemic control.” These reflections highlight the interpersonal nature of deprescribing conversations and the limitations of nudges that do not account for the dynamics of shared decision-making.

### Association Between Response to OPAs and Characteristics

We first ran a logistic regression to evaluate whether there was an association between a provider acting upon an OPA (1/0) and the characteristics of the OPAs and encounters (the context in which OPAs are responded to). We also assessed whether the provider commented on the OPA and whether the valence of the comment (negative versus positive) was related to these characteristics. This analysis aimed to identify structural or contextual features that influence not only the likelihood of providers acting on nudges but also how they engaged with and reacted to the CDS tool.

From the intercept, providers had a 0.61% chance of acting through the OPA in an encounter (OR = 6.13E-03, SE = 1.47, 95% CI = [2.89E-03, 1.30E-02]). This low base rate reflects the general rarity of deprescribing behavior in response to the CDS nudges. For every extra OPA fired during an encounter, a provider was 53.1% less likely to take the recommended action (OR = 0.469, SE = 1.14, 95% CI = [0.365, 0.603]). This suggests that a higher volume of nudges may contribute to alert fatigue or reduce the perceived salience of each prompt.

When commenting through an OPA, providers were approximately three times more likely to leave a negative comment than a positive one (OR = 3.28, SE = 1.28, 95% CI = [2.04, 5.29]). This asymmetry in comment valence may reflect provider skepticism toward the guideline, frustration with workflow disruptions, or disagreement with the timing or content of the nudge. Other encounter-level characteristics, such as patient age, provider specialty, and visit type, were not significantly associated with the valence of provider action or comment (see ST1), suggesting that broader contextual and design-related features may have had a more substantial impact. Together, these findings highlight how both nudge saturation and provider perceptions may influence the effectiveness of CDS.

**Figure 2:**
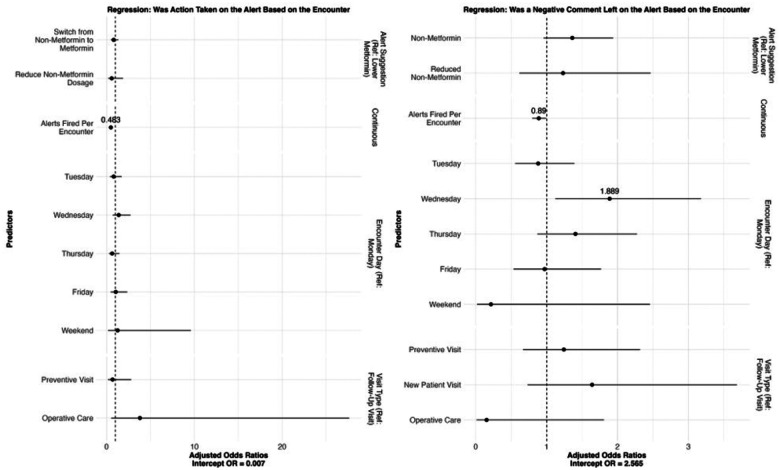
Odds ratios on associations between providers leaving comments on OPAs (left) or providers leaving negative comments (right) and encounter characteristics. Significant odds ratios have values labeled.

Odds plots can be found in Figure 2, and computed odds ratios can be found in Supplement Tables (STs) 1 and 2.

A multinomial logistic test was conducted to determine whether an identified rejection theme (versus acceptance of the OPA) was associated with the type of glycemic control rejected or a patient’s mortality, based on the premise that provider preferences may vary by clinical scenario. We hypothesized that different nudges (e.g., suggestions to reduce insulin versus discontinue sulfonylureas) might elicit distinct reasons for rejection, reflecting variation in provider decision-making. The null hypothesis was that no significant association exists between the reason a provider rejected a recommendation and the type of glycemic control targeted. The alternative hypothesis was that rejection themes were significantly associated with specific types of medication recommendations, suggesting that how providers respond to CDS is not uniform but influenced by the clinical content of the nudge.

**Figure 3:**
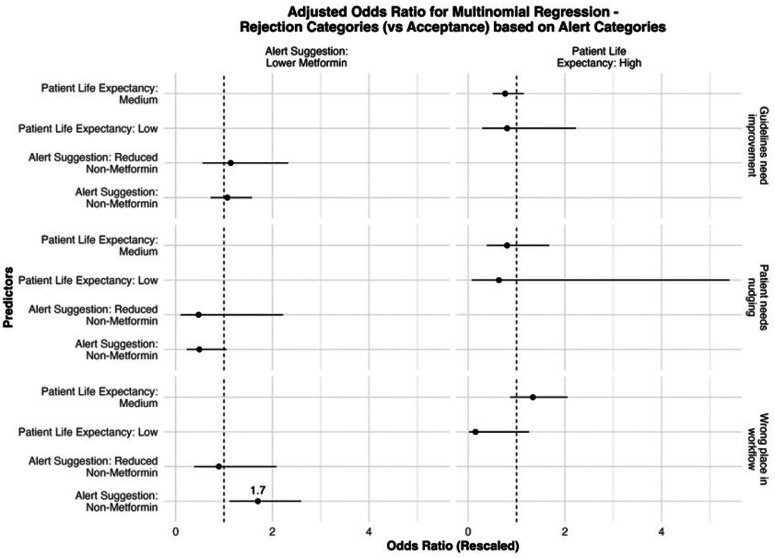
Multinomial logistic regression to evaluate the association between rejection and OPA themes.

Compared to agreeing with the OPA-suggested CW guidelines, providers were 31% more likely to comment that the guidelines need improvement (OR = 1.31, SE = 1.08, 95% CI = [1.13, 1.53]). This suggests that disagreement with the clinical recommendation itself—rather than external factors—was a common reason for rejecting the nudge. However, providers were 62.6% less likely to comment that the OPA was in the wrong place in the workflow than to agree with the guideline (OR = 0.374, SE = 1.16, 95% CI = [0.280, 0.499]). This indicates that perceived misalignment with workflow, while important, was a less frequent basis for provider rejection than guideline disagreement. Commenting about the inappropriate placement of the OPA in the workflow was less likely when the patient’s estimated life expectancy was low (OR = 0.427, SE = 1.28, 95% CI = [0.265, 0.688]). This may reflect a greater willingness to consider deprescribing in patients with limited life expectancy, regardless of the timing of the nudge. If a provider commented that a patient needed nudging, it was less likely when their life expectancy was moderate compared to high (OR = 0.745, SE = 1.11, 95% CI = [0.604, 0.918]). This may suggest that providers anticipate more resistance to deprescribing in patients who appear healthier or are expected to live longer. Results are presented in Figure 3 and ST3.

### Representativeness Checks

**Figure 4:**
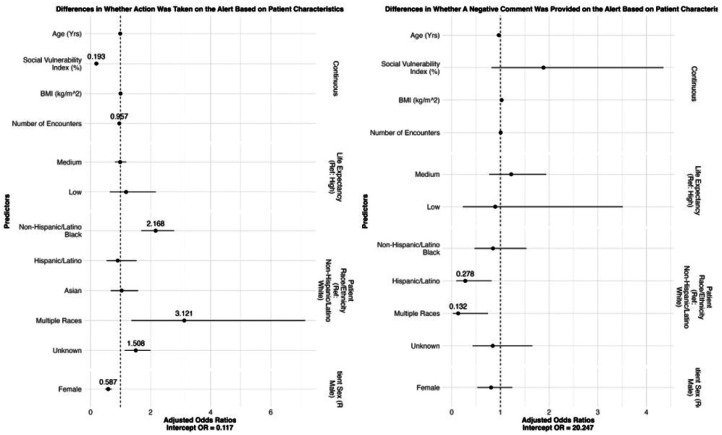
Odds ratios on associations between providers leaving comments on OPAs (left) or providers leaving negative comments (right) on patient characteristics.

**Figure 5:**
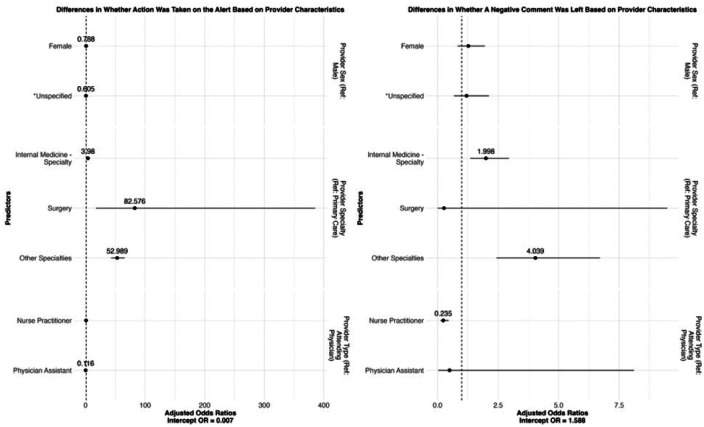
Odds ratios on associations between providers leaving comments on OPAs (left) or providers leaving negative comments (right) on provider characteristics.

We conducted three checks to assess whether the populations engaging with OPAs through comments were demographically representative of the individuals featured in the OPAs. Results are presented in Figure 4 (patients) and Figure 5 (providers) and ST4, 5, 6, and 7. First, we used logistic regression to determine whether comments on OPAs were left by a representative population of physicians and a representative population of patients. If comments were left on an OPA, we evaluated whether the valence of the comment (i.e., whether it was positive or negative) was related to the characteristics of the provider or the patient.

Compared to male providers, female providers were 21.9% less likely to leave a comment on an OPA (OR 0.788, SE = 1.08, 95% CI = [0.652, 0.954]). Providers were 59% more likely to leave a negative comment than a positive one (OR = 1.59, SE = 0.18, 95% CI = [1.15, 2.19]), which reinforces earlier findings that commentary tends to reflect disagreement or workflow issues. Specialty providers were significantly more likely to leave comments—and particularly negative comments—than general internal medicine providers, indicating differences in CDS response patterns by clinical context or expertise.

Female patients were 41.3% less likely to have comments left on their OPA fires compared to male patients (OR = 0.587, SE = 1.09, 95% CI = [0.492, 0.701]), though not significantly more negative comments (OR = 0.808, SE = 1.25, 95% CI = [0.525, 1.24]). Some racial and ethnic minorities, compared to non-Hispanic/Latino White patients, were more likely to have comments left on their OPA firings. However, whether negative comments were left was not necessarily significantly different. Patients of higher social vulnerability were 80.67% less likely to have a comment left on their OPA firing (OR = 0.193, SE = 1.19, 95% CI = [0.138, 0.270]), and patients who had more encounters with their provider had a slightly less chance of having comments left on their OPA firing (OR = 0.957, SE = 1.01, 95% CI = [0.945, 0.970]), potentially reflecting **greater reliance on clinical familiarity over CDS prompts in ongoing care relationships**.

**Figure 6:**
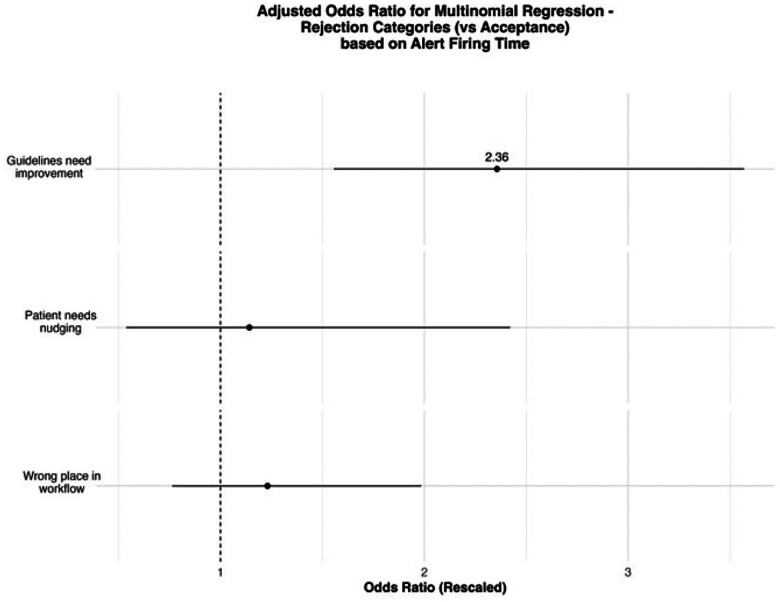
Multinomial regression of themes identified in the comments and OPA firing time.

We used a multinomial logistic regression to compare whether the negative themes (compared to the positive theme of the comments left on OPAs during the RCT period) represented the negative themes of the comments left during the entire period of the OPAs’ firings. The most significant difference was that providers were more likely to indicate the OPA was in the wrong place in the workflow during the RCT period than during the RCT period. Otherwise, no significant differences were detected, and the one significant baseline comparison (indicating a wrong placement in the workflow compared to approval) was consistent with the results seen in the multinomial logistic regression of Figure 3, indicating the stability of the results. Results are presented in Figure 6 and ST8.

## DISCUSSION

We conducted a mixed-methods evaluation to identify potential reasons for the ineffectiveness of nudges within the EHR system in promoting CW guidelines among older adults with DM2 in a large academic health system. We help fill a gap in the existing literature that examines the acceptability of implementing deprescribing interventions.(26)

Three major themes from the comments reveal barriers to de-implementation. First, de-implementation guidelines need to be specific and clear. The CW guidelines require additional information to increase confidence in them and facilitate structured decision-making regarding glycemic control while allowing for physician autonomy. For example, more robust alternate measures of identifying glycemic control, including when medications are used for non-DM2 reasons and when comorbidities or contraindications come into play, can help providers understand how CW guidelines apply to their patients. The CW guidelines, which informed the CDS design and were algorithmically integrated into the EHR, need to provide explainability support to increase concordant decision-making by providers. Evidence has shown that individuals are less likely to trust algorithm-based decisions when the algorithmic suggestions go against their decision-making in similar contexts. (27)

Second, despite the provider’s efforts to prescribe medications and educate the patient, the comments identified that the patient needs support outside of the provider interaction to be empowered to change their medications. There are active efforts within behavioral economics intervention designs to “boost” patients (i.e., combining empowerment with nudging to promote the motivation underlying action). (28) This is critical when guidelines are based on, to a patient, the disturbing notion of their mortality. The specific CW guidelines made salient information that the patient could fundamentally disagree with (e.g., specific patient complexities around hypoglycemia for physicians or impending mortality for patients). One possible solution to improve patient confidence could be to nudge patients independently of their providers to promote discussion about best care practices. (13) Another potential solution to mitigate patient hesitancy would be to integrate shared decision-making tools into the EHR that are tailored to each patient’s expertise, such as their lived experiences. These tools can help providers and patients reach agreement on making difficult healthcare decisions and acting on guideline recommendations by providing structured guidance and helping them weigh priorities in healthcare.(29)

Third, nudges were in the wrong place within the workflow. One, the OPAs were not fired appropriately within the EHR as per the decision-making for medication prescriptions. Providers who were not the primary point of contact for the patient’s DM2 diagnosis were nudged, leading to inaction. Engineering of CDS to focus on the point-of-contact provider could alleviate alert fatigue and increase the success rate of CDS. Two, the CDS could be easily ignored by the provider. Nudges require thoughtful design and integration into other healthcare implementations to ensure their effectiveness, as alert fatigue and clinician desensitization to CDS can lead to the overlooking of highly important CDS. Especially for CDS focused on de-implementing harmful practices, it is much harder to stop ingrained practices than to introduce new ones, which is a focus of most behavioral economics and implementation science research. (30) Further research can explore how EHR metadata, which describes the granular interactions with the EHR, can inform CDS design into clinical workflows to get provider attention for intended behavior change.(31)

The analyses have limitations. First, the OPAs’ acknowledgment rate was low. Second, given that very few providers left comments within the OPAs, statistical significance can be attributed to noise rather than being representative of the provider population. Further analyses of misplaced or underutilized CDS in an EHR could explore multiple contexts, incorporating variables such as inpatient versus ambulatory care and other disease types to develop a unified tool that evaluates problematic CDS. (32) We also acknowledge that we identified the ineffectiveness of the CW guidelines in one clinical setting, disease, and population. The exact results may not be directly applicable to other clinical settings. Still, the methods and data can contribute to the broader research scopes of nudging effectiveness, health services research, and implementation science.

## CONCLUSION

This study demonstrates that EHR comments on CDS can provide valuable evidence regarding how nudges may not affect meaningful clinical behavior changes. We identified key themes surrounding provider resistance to reminders and assessed whether the lack of responsiveness was related to encounter, CDS, patient, or provider characteristics. These contextual variables inform better tailoring of CDS. We contribute to the implementation science literature by offering a method for CDS evaluation to enhance its implementation for healthcare delivery. Other studies that have implemented nudges within the EHR have shown minimal effects on changing provider behaviors toward patients. The comments shed light on why the nudges in this randomized trial were ineffective at significantly altering prescriber behavior.

## Supplementary Files

This is a list of supplementary files associated with this preprint. Click to download.

- Additionalfile2.docx
- NudgeBudgeSupplement.xlsx
- SUPPLEMENTALTABLES.docx

## Figures and Tables

**Table 1:** Descriptive Statistics of Patients and Providers Based on Comments Left or Not

	Any Alert Firings	Alert Firings Without Acknowledgment Comments	Alert Firings With Acknowledgment Comments	Alert Firings Without Acknowledgment Comments (During RCT)	Alert Firings With Acknowledgment Comments (During RCT)
Patient Characteristics					
Unique Patients	7663	7610 (92.6%)	564 (7.4%)	4308 (56.2%)	197 (2.6%)

Female	3986 (52.7%)	3965 (52.7%)	267 (47.7%)	2297 (53.2%)	93 (47.0%)
Male	3583 (47.3%)	3552 (47.2%)	293 (52.3%)	2020 (46.8%)	105 (53.0%)
Other/Unknown	1 (<0.1%)	1 (<0.1%)	0 (0%)	1 (<0.1%)	0 (0%)

Non-Hispanic/Latino White	5339 (69.7%)	5303 (69.7%)	381 (67.6%)	3124 (72.5%)	140 (71.1%)
Non-Hispanic/Latino Black	879 (11.5%)	874 (11.5%)	70 (12.4%)	466 (10.8%)	24 (12.2%)
Asian	336 (4.38%)	336 (4.42%)	22 (3.9%)	186 (4.32%)	5 (2.54%)
Hispanic/Latino	308 (4.02%)	308 (4.05%)	29 (5.14%)	150 (3.48%)	8 (4.06%)
Multiple Races	31 (0.405%)	30 (0.394%)	4 (0.709%)	23 (0.534%)	1 (0.508%)
Native American/Native Alaskan/Native Hawaiian	15 (0.196%)	15 (0.197%)	0 (0%)	8 (0.186%)	0 (0%)
Other Race/Ethnicity	8 (0.104%)	8 (0.105%)	1 (0.177%)	1 (0.0232%)	0 (0%)
Unknown	748 (9.76%)	737 (9.68%)	57 (10.1%)	350 (8.12%)	19 (9.64%)

Age - years (Mean (SE), Median [IQR])	81.4 (0.0423) 80.2 [77.2, 84.3]	81.4 (0.0424) 80.2 [77.2, 84.3]	81.8 (0.192) 80.8 [78, 84.6]	82.1 (0.0541) 81.1 [78.1, 85]	82.2 (0.337) 81.4 [78.1, 85]

Social Vulnerability Index (Mean (SE),Median [IQR])	0.518 (0.00246) 0.484 [0.272, 0.798]	0.518 (0.00246) 0.489 [0.272, 0.798]	0.433 (0.011) 0.41 [0.195, 0.646]	0.509 (0.00325) 0.475 [0.249, 0.798]	0.397 (0.0186) 0.382 [0.161, 0.572]

Patient BMI (Mean [SE], Median [IQR])	29.4 (0.422) 28.3 [25.2, 32.3]	29.4 (0.424) 28.3 [25.2, 32.3]	28.8 (0.249) 28 [24.9, 32.3]	29.1 (0.0718) 28.4 [25.2, 32.6]	29 (0.417) 28.6 [25, 32.9]

Number of Encounters per Patient (Mean[SE], Median [IQR])	5.35 (0.0497) 3 [2, 7]	5.38 (0.0498) 3 [2, 7]	7.05 (0.273) 5 [2, 9]	7.26 (0.0765) 5 [3, 9]	7.68 (0.45) 6 [3, 10]

Provider Characteristics					
Unique Providers	365	364	82	275	40

Female	137 (37.5%)	137 (37.5%)	28 (34.1%)	110 (40%)	14 (35%)
Male	117	117	31	96	13
Other/Unknown	111	110	23	69	13

Physician (MD or DO)	289 (77.3%)	288 (77.2%)	69 (84.1%)	220 (80.292%)	35 (87.5%)
Nurse Practitioner (NP)	65 (17.4%)	65 (17.4%)	10 (12.2%)	40 (14.5985%)	5 (12.5%)
Physician Assistant (PA)	18 (4.81%)	18 (4.83%)	3 (3.66%)	13 (4.74453%)	0 (0%)
Registered Nurse (RN)	1 (0.267%)	1 (0.268%)	0 (0%)	1 (0.364964%)	0 (0%)

Internal Medicine - General	268 (71.7%)	268 (71.8%)	61 (74.4%)	197 (71.9%)	26 (65%)
Internal Medicine - Specialty	88 (23.5%)	87 (23.3%)	18 (22%)	66 (24.1%)	13 (32.5%)
Other Specialties	16 (4.28%)	16 (4.29%)	2 (2.44%)	10 (3.65%)	1 (2.5%)
Surgery	3 (0.802%)	3 (0.804%)	1 (1.22%)	1 (0.365%)	0 (0%)

Encounter Characteristics					
Unique Encounters	39654	39132	763	13791	207
Alerts Fired Per Encounter (Mean [SE], Median [IQR])	3.05 (0.00834) 3 [2, 4]	3.07 (0.0084) 3 [2, 4]	2.3 (0.0538) 2 [1, 3]	2.85 (0.013) 3 [2, 4]	1.96 (0.0805) 2 [1, 2]

Follow-Up Visit	34954 (88.1%)	34501 (88.2%)	657 (86.1%)	12254 (88.9%)	187 (90.3%)
New Patient Visit	1126 (2.84%)	1103 (2.82%)	40 (5.24%)	362 (2.62%)	9 (4.35%)
Preventive Visit	2588 (6.53%)	2543 (6.5%)	62 (8.13%)	878 (6.37%)	10 (4.83%)
Operative Care	188 (0.474%)	188 (0.48%)	3 (0.393%)	48 (0.348%)	0 (0%)
Other	386 (0.973%)	386 (0.986%)	0 (0%)	50 (0.363%)	0 (0%)
Procedure Visit	203 (0.512%)	202 (0.516%)	1 (0.131%)	67 (0.486%)	1 (0.483%)
Sick Visit	207 (0.522%)	207 (0.529%)	0 (0%)	131 (0.95%)	0 (0%)
Telemedicine Visit	2 (0.00504%)	2 (0.00511%)	0 (0%)	1 (0.00725%)	0 (0%)

Monday	7761 (19.6%)	7650 (19.5%)	167 (21.9%)	2724 (19.8%)	39 (18.8%)
Tuesday	9112 (23%)	8992 (23%)	179 (23.5%)	3290 (23.9%)	45 (21.7%)
Wednesday	8348 (21.1%)	8250 (21.1%)	158 (20.7%)	2858 (20.7%)	60 (29%)
Thursday	8297 (20.9%)	8162 (20.9%)	182 (23.9%)	2841 (20.6%)	44 (21.3%)
Friday	5553 (14%)	5498 (14%)	74 (9.7%)	1906 (13.8%)	19 (9.18%)
Weekend	582 (1.47%)	579 (1.48%)	3 (0.393%)	172 (1.25%)	0 (0%)

Alert Characteristics					
Number of Alert Firings	67412	66633	779	24830	215

Patient Life Expectancy: High	40329 (60.8%)	39821 (60.7%)	508 (66.5%)	15381 (63.9%)	153 (73.9%)
Patient Life Expectancy: Medium	24441 (36.8%)	24204 (36.9%)	237 (31%)	8126 (33.8%)	51 (24.6%)
Patient Life Expectancy: Low	1564 (2.36%)	1545 (2.36%)	19 (2.49%)	559 (2.32%)	3 (1.45%)

Alert Suggestion: Lower Metformin	35474 (53.5%)	35035 (53.4%)	439 (57.5%)	13015 (54.1%)	133 (64.3%)
Alert Suggestion: Switch from Non-Metformin to Metformin	26669 (40.2%)	26395 (40.3%)	274 (35.9%)	9194 (38.2%)	58 (28%)
Alert Suggestion: Reduced Non-Metformin	4191 (6.32%)	4140 (6.31%)	51 (6.68%)	1857 (7.72%)	16 (7.73%)

No Action Taken Through Alert	67330	66561	769	24793	213
Action Taken Through Alert	82	72	10	37	2

**Table 2:** Main Themes, Subthemes, and Representative Comments during the entire OPA Activation Period.

Theme	The CW guidelines need improvement	The patient needs nudging.	OPA is in the wrong place in the workflow.	Provider utiliz
Number of Comments (N Comments = 565)	308 (54.5%)	69 (12.2%)	203 (36.0%)	208 (36.8%)
Sub-themes	No symptoms or doing well, so no reason to take action. (N = 160) Clinician doesn’t agree with nudge or guidelines. (N = 78) Other clinical reason to not take action. (N = 76) A1c is secondary objective (e.g., primary weight loss, etc.) (N = 9)	N/A	Followed by another clinician (e.g., PCP, endocrinologist) so not their responsibility (N = 125) Awaiting new lab result/will re-evaluate. (N = 89)	N/A
Representative Comments		“patient refuses to reduce meds”	“await labs”	
	“patient with hyperglycemia with BS at home 200–400” “look at his glucose; these prompts are silly”			Metformin st will continue Prandin in th taper off
	doing well with low dose metformin which poses no risk	“Patient has been reluctant to stop his medication despite the guidelines which have been discussed and he clearly understands however since he is tolerating his regimen wel l with no hypoglycemic episodes he asks to continue treatment.”	“DM followed by PCP”	dose lowered daily.
	“multiple co-morbities with vascular disease and CKD which require better glycemic control”			meds reduce
	“CVD secondary prevention.”	“Patient wants to stay on medication optimize glycemic control”	“Will consider at next appointment”	

Number of unique providers providing this type of comment	59	27	41	47
Number of unique patients for this type of comment	260	60	150	189

OPA firings (N)				
High Life Expectancy OPAs	219	46	120	136
Medium Life Expectancy OPAs	80	30	82	65
Low Life Expectancy OPAs	9	3	1	7

OPA Suggestion: Lower Metformin	183	50	96	123
OPA Suggestion: Switch from Non-Metformin to Metformin	103	15	93	70
OPASuggestion: Reduced Non-Metformin	22	4	13	15

## Data Availability

Deidentified data may be available upon request.

## Abbreviations

A1C: Hemoglobin A1c
ABIM: American Board of Internal Medicine
CAD: coronary artery disease
CDS: Clinical Decision Support
CKD: Chronic Kidney Disease
CW: Choosing Wisely Guidelines
DM2: Diabetes mellitus type 2
EHR: electronic health record
IQR: interquartile range
NYU: New York University
OPA: Our Practice Alert
OR: Odds ratio
RCT: randomized controlled trial
SEM: standard error of the mean
ST: supplemental table
